# Increased Risk of the *APOB* rs11279109 Polymorphism for CHD among the Kuwaiti Population

**DOI:** 10.1155/2017/6963437

**Published:** 2017-12-06

**Authors:** Suzanne A. Al-Bustan, Fatma G. Ismael, Ahmad Al-Serri, Ibrahim Al-Rashdan

**Affiliations:** ^1^Department of Biological Sciences, Faculty of Science, Kuwait University, Kuwait City, Kuwait; ^2^Department of Pathology, Human Genetics Unit, Faculty of Medicine, Kuwait University, Kuwait City, Kuwait; ^3^Department of Medicine, Faculty of Medicine, Kuwait University, Kuwait City, Kuwait

## Abstract

**Background:**

Coronary heart disease (CHD) is among the leading causes of death in Kuwait. This case-control study investigated the genetic association of *APOB* rs11279109 with CHD in Kuwaitis.

**Methods:**

The polymorphism was genotyped in 734 Kuwaiti samples by direct amplification. Statistical analysis with genetic modeling was used to assess its association with CHD.

**Results:**

A statistically significant association (*P* < 0.001) between the rs11279109 *DD* genotype (OR: 2.43, CI: 1.34–4.41) with CHD was observed. A codominant genetic model revealed a 2.69 risk increase (CI: 1.57–4.61) for the *DD* genotype (*P* = 0.009) independent of age, sex, BMI, smoking, hypercholesterolemia, and ethnicity suggesting *APOB* rs11279109 as an indicator for the increased risk of CHD.

**Conclusion:**

The *DD* genotype may explain molecular mechanisms that underline increased LDL oxidation leading to arthrosclerosis. The findings emphasize the need to identify genetic markers specific to the CHD patient ethnic group in order to improve prognosis and help in early diagnosis and prevention.

## 1. Background

Coronary heart disease (CHD) is the leading cause of death and disability-adjusted life years (DALYs) worldwide [1] accounting for about 39.5% of all deaths in Kuwait [2, 3]. CHD results from poor circulation of blood and oxygen as a result of the narrowing of the small blood vessels. The manifestation of CHD is usually presented with myocardial infarction and/or angina, due to coronary atherosclerosis. Extensive studies [4, 5] have demonstrated that the interaction between various environmental factors and specific genetic polymorphisms leads to CHD. However, the exact etiology and molecular mechanisms remain unresolved. The association of advanced age, sex, nutrition, smoking, metabolic syndrome, and physical inactivity has been studied extensively and documented in the Kuwaiti population [2, 6, 7]. Limited studies have focused on the association of genetic factors with CHD in either the Kuwaiti population [8–10] or among Arab ethnic groups in general [11].

During the past decades, genetic association studies and genome-wide association studies have focused on the identification and characterization of genetic polymorphisms that may increase the susceptibility to CHD in the presence of one or more risk factors and in specific ethnic groups and/or populations [12–19]. The highly implicated risk factors for CHD pathogenesis are increased levels of cholesterol and low-density lipoproteins (LDL) and/or decreased levels of high-density lipoproteins (HDL). Lipids are well known to play a major role in the development of type 2 diabetes mellitus (T2DM), obesity, and hypertension, collectively known as the metabolic syndrome that subsequently could result in heart disease. Therefore, it is important to characterize genes responsible for lipid transport and metabolism including the Apolipoprotein family. Mutations in these polymorphic genes may alter the protein function thereby affecting the transport and metabolism of lipoproteins. Such variants may also interact with common risk factors leading to atherosclerosis and consequently to the manifestation of CHD. One of these genes is *APOB*.

There are two forms of the ApoB protein, apoB-100 and apoB-48, resulting from tissue-specific alternative splicing. ApoB-100 consists of 4560/4563-amino acids including a 27-residue signal sequence. The mature protein forms part of chylomicrons and very low-density lipoproteins (VLDL) that are synthesized during lipoprotein metabolism; in addition, it contains the binding site for the uptake of LDL by its receptor [20]. The human *APOB* gene, localized to chromosome 2p23-24, is 43 Kb comprising 29 exons and 28 introns [21]. Numerous polymorphisms at the *APOB* gene locus have been reported and studied [19, 22–26]. One commonly studied polymorphism is an insertion/deletion variant (rs11279109) within the promoter region coding for the signal peptide. There are two common alleles: an insertion allele (I, sp27) which codes for the 27-amino acid signal peptide and a deletion allele (D, sp24) which codes for the 24-amino acid signal peptide lacking the hydrophobic leucine-alanine-leucine residues [27]. The most commonly reported genotype is II with variable frequencies in different populations [28–31].

Conflicting results have been reported with regard to the association of this polymorphism with CHD, and very limited studies have been reported in the Kuwaiti population [8–10]. Therefore, this study aimed to analyze the genetic association of *APOB* rs11279109 with the increased risk of developing CHD. The Kuwaiti population is heterogeneous with two major ethnic groups, Arabs including Bedouin Arabs and Persians, while the rest are an admix [26]. In this paper, genetic association of *APOB* rs11279109 with the increased risk to CHD in Kuwaiti Arabs is reported.

## 2. Methods

### 2.1. Sample Description and Diagnostic Criteria

Blood samples were collected from a total of 734 Kuwaiti participants including 371 CHD patients and 363 controls that were matched as closely as possible based on age and sex. Patient's samples (*n* = 371) were obtained from patients who were routine visitors to the outpatient clinics for follow-ups at Kuwait Chest Hospital, Al-Amiri Hospital, and Mubarak Hospital. The CHD patients included 246 males and 126 females with a mean age of onset of CHD of 48.4 years. Clinical diagnosis was provided from the medical records. The inclusion criteria were based on the medical history of the presence of typical chest pain, echocardiogram and previous history of MI, coronary angioplasty, or percutaneous transluminal coronary angioplasty (PTCA), and coronary artery bypass grafting (CABG). The controls (*n* = 363) consisted of a random sample of Kuwaiti patients who were visiting the hospital for a routine checkup and had medical records. The inclusion criteria were being devoid from any type of cardiovascular diseases and having a normal coronary artery with no evidence of plaque or peripheral vascular disease as confirmed by angiography, medical profile, or previous medical history as documented in their medical records. The controls were matched based on sex and age (±2 years) and included 228 males and 135 females with a mean age of 54.2 years. This study has been approved by the local ethical committee at Kuwait University as well as the Ministry of Health Ethical Board, Kuwait. The sample and medical data collection protocol and informed consents used were in accordance to the modified Helsinki guidelines of 1975 and revised in 2000. Informed consent from each participant in this study was obtained.

### 2.2. Assessment of Risk Factors to CHD

For each sample, information regarding age, age of onset of CHD, medical history of metabolic disorders, lipid profile, BMI (calculated as body weight (kilogram) divided by height (meter) squared (kg/m^2^)), smoking, and family history were documented with a standardized questionnaire for each patient as well as for each of the control samples. Medical history for hypertension, dyslipidemia, and T2DM were documented based on medical records, medication, and filed reports. In addition, ethnicity was documented based on recording parental origins both paternally and maternally dating back at least four generations. Informed consent was obtained from all the volunteers. All data was logged into the SPSS software (Version 22; SPSS Inc., Chicago, IL, USA) for statistical analysis and association to CHD and *APOB* rs11279109 polymorphism. Modifiable (BMI, dyslipidemia, T2DM, hypertension, and smoking) and nonmodifiable factors (age, sex, family history, and ethnicity) were assessed for their contribution to CHD in the sampled population. No significant differences in the documented phenotypes were observed between males and females.

### 2.3. Genotyping *APOB* rs11279109 Polymorphism

From 5 ml of whole blood, total genomic DNA was isolated with proteinase K digestion and salting out [32]. The rs11279109 polymorphism was analyzed by direct amplification of a 93 bp target sequence employing the polymerase chain reaction (PCR) as described previously [31, 33]. A 4% 3 : 1 Nusieve: agarose gel prestained with ethidium bromide (10 mg/ml) was used to resolve the PCR products by electrophoresis for one hour at 200 V and 100 mA. Bands were visualized under UV and documented using Syngene Digital documentation system (Synoptics Ltd., UK), and the fragment size of the products was determined by comparison to a 123 bp DNA ladder using Gene tools software (version 4.00). Different genetic models based on the obtained genotypes were devised.

### 2.4. Statistical Analysis

Results were expressed as mean ± SEM and percentages where appropriate. Nonparametric analysis was performed where appropriate. Logarithmic transformation was applied where appropriate. Hardy-Weinberg equilibrium (HWE) was tested using the web-based calculator available at http://www.tufts.edu/, which confirmed the population to be in equilibrium. The genetic association was analyzed and controlled for age, sex, BMI, subethnicity, smoking status, and total cholesterol using the SNPassoc package from R software (R Stats Package, Version 3.3.0). The results are expressed as odds ratio (OR) with 95% confidence intervals (CI). All other statistical analyses were performed using SPSS software (Version 22; SPSS Inc., Chicago, IL, USA). Power calculation on the sample size was estimated using the statistical software package StatCalc (version 7.1.2.0; Epi InfoTM, Atlanta GA, USA). A power > 80% at alpha = 0.05 was achieved in this study assuming an average OR of 2.5 and minor allele frequency of 7%.

## 3. Results

### 3.1. Assessment of Risk Factors to CHD

Modifiable and nonmodifiable (age, sex, ethnicity, and family history) risk factors of the CHD patients (*n* = 372) compared with those of the control group (*n* = 363) are presented in Table 1. No significant differences in the frequency distribution of sex, age, and BMI between the CHD patients and their controls were observed. Significant differences in the ethnicity of the two groups were observed in which the percentage of Kuwaiti patients of Arab ancestry was significantly higher (*P* < 0.001) among the CHD patients than the controls.

### 3.2. Genetic Association of *APOB* rs11279109 with the Association of Common Risk Factors and CHD

The most frequent genotype for *APOB* signal peptide polymorphism was the *II* genotype: 51.9% for the CHD patients and 60.9% for the controls (Table 2). However, the frequency of the *D* allele in the CHD patients (0.32) was found to be higher than that of the *I* allele (0.68) as well as higher than that in the controls where the frequency of the *D* allele was 0.23. Genotype and allele frequencies for the rs11279109 were found to be in HWE (*P* > 0.05) for the controls; however, a significant deviation (*P* = 0.015) was observed in the CHD group (*n* = 372) in which the proportion of the *DD* genotype was found to be higher in Arab Bedouins (21.2%) as compared to the other groups (Table 3). Genotype distribution for rs11279109 for all other risk factors assessed showed no significant differences (*P* > 0.05).

Multivariate logistic regression analysis, in which CHD was the dependent variable and the independent variables were the environmental risk factors, was used to investigate the contribution of the *DD* genotype in the presence of the common risk factors to increase the risk to CHD in Kuwaitis and to assess their ORs. A summary of the results is presented in Table 4. The significant predictors of CHD in this model were smoking status (*P* < 0.001), medical history of diabetes mellitus (*P* < 0.001), high cholesterol level (*P* = 0.002), and family history of cardiac diseases (*P* = 0.024) after adjusting for the confounding variables (age and sex). The chance of having CHD was estimated to be 6.69 times higher for patients who ever smoked than those who never smoked, 5.73 times more for patients having positive medical history of diabetes mellitus, 1.85 times more for patients with positive medical history of high cholesterol, and 1.69 times higher among patients having positive family history of cardiac diseases than those who did not. Statistically significant associations with increased odd ratios for CHD was also observed for the *APOB* rs11279109 polymorphism in which an OR = 2.43 was observed for the *DD* genotype.

### 3.3. Genetic Modeling of *APOB* rs11279109 and CHD

Four different genetic models (Table 5) were devised to demonstrate the independent association of the *APOB* rs11279109 polymorphism with CHD in Kuwaiti patients after adjusting for sex, BMI, and as well as the identified common risk factors in this study including ethnicity, smoking status, and medical history of hypercholesterolemia. Due to missing data in total cholesterol and BMI (*n* = 67) in some CHD patients and controls, the number of samples analyzed in these models was 336 in CHD patients and 332 controls. A statistically significant association of the *D* alleles and *DD* genotype remained significant (*P* < 0.01) in four genetic models implicating its status as a “risk” allele. Only the over-dominant model was nonsignificant (*P* > 0.05) for the *DD* genotype. The highest OR ratio of 2.69 (*P* = 0.0009) for the *DD* genotype was observed in the codominant model.

## 4. Discussion

This study extensively analyzed known risk factors for CHD in addition to a commonly studied genetic polymorphism, *APOB* rs11279109, among a sample of Kuwaiti natives (*n* = 735). The results from this study confirmed and further characterized previously reported risk factors in addition to newly identified risk factors and estimated their risk in the CHD patients (*n* = 372) sampled from the Kuwaiti population. In the current study, the observed statistically significant association between smoking (*P* < 0.001), hypocholesteremia (*P* < 0.001), and hypertriglyceridemia (*P* < 0.01) with CHD is in agreement with other studies reported for different populations [19, 24, 34–39].

The statistically significant association of ethnicity (*P* < 0.001) and the known risk factors (*P* < 0.005), such as smoking (32.3%; OR of 6.69), hypertension (55.1% of CHD patients), medical history of T2DM (53.1%; OR of 5.73), hypercholesterolemia (OR of 1.85), and family history of CHD (OR of 1.69) in addition to the newly identified *APOB* rs11279109 *DD* genotype (OR of 2.43), all contribute to the increased risk of CHD possibly through the development of different mechanisms leading to arthrosclerosis as a result of LDL oxidation. Increased cholesterol and TG levels have been shown to lead to the development of fibrotic plaques within the walls of the arteries [40] and to increase amounts of atherogenic lipoproteins such as LDL particles [41] and smaller TG-rich lipoproteins [42]. Although no statistically significant association (*P* > 0.05) was observed with regard to dyslipidemia and increased risk to CHD in this study, the 1.88-fold increase of hypercholesterolemia and the higher statistically significant association of the *DD* genotype at the *APOB* signal peptide locus may implicate the role of this polymorphism in increasing the risk to CHD independently. It has been suggested that the *APOB* rs11279109 *DD* genotype results in a 66% reduction in the translocation of the *APOB*-100 from the endoplasmic reticulum along with the increased hydrophobicity [33] that may result in slower transfer of the *APOB*-100 and formation of LDL particles. The slow transfer and possible increased deposition of LDL due to a diet high in fat may cause an accumulation of LDLs in the vessels subjecting them to increased oxidation. No statistically significant association (*P* > 0.05) was found between the *DD* genotype and cigarette smoking despite its 6-fold increase in the risk to CHD among the studied population. However, the statistically significant association (*P* < 0.001) of the *DD* genotype (OR 2.69) remained after adjusting for moking suggesting independent contribution from both risk factors in the mechanism of developing arthrosclerosis. Hypertension may also contribute to this mechanism through the pathophysiologic mechanisms that link hypertension with lipids and other metabolic disorders that eventually lead to CHD [43]. Despite the lack of association between the *APOB* rs11279109 with hypertension and dyslipidemia in this study, the role of other polymorphisms at the *APOB* gene locus or other loci may be contributing to this pathogenesis [19]. T2DM was demonstrated to be a significant risk factor (OR 5.73) for CHD which is in agreement with several studies that compared the CHD risk in T2DM patients as compared with nondiabetic subjects [8, 19, 44].

Several studies have indicated that a positive family history of cardiac disease increases the risk of CHD [9, 45, 46]. In the present study, 27.7% of CHD patients had a positive family of cardiac diseases. Statistical analysis revealed a highly statistically significant association between CHD patients and positive family history of cardiac disease (*P* = 0.002), and the risk of CHD is 1.67 times higher among individuals having positive family history of cardiac diseases compared to those with no family history. Myers and others found that the association between positive family history and CHD reflects either shared genetic predisposition or shared environmental habits [45]. This also applies to the studied population in this cohort as the majority of the samples were of Arab Bedouin or Persian ancestry (Table 1). Both these ethnic groups have similar cultural practices of consanguinity and nutrition high in fat. Ethnicity was also found to be significantly (*P* < 0.001) associated with CHD among the sampled Kuwaiti population, particularly among those from Bedouin Arab (*n* = 95, 25.5%) and Iranian origins (*n* = 97; 26.1%) in comparison to the control group. In addition, HWE was deviated with regard to genotype distribution in the patient group only in which the homozygote DD genotype was in excess for the Arab Bedouin ethnic group (21.2%, *P* = 0.015). These findings suggest that due to consanguinity the frequency of the D allele increased in the gene pool of these ethnic groups. In addition to other cultural practices, nutrition known to be very high in fat, differences in the structure of the LDL particles, and increased frequency of the *DD* genotype at the *APOB* locus increased the risk for CHD in Kuwaiti Arab Bedouins. However, the association of *APOB* rs11279109 polymorphism, with increased risk for CHD, remained significant (*P* < 0.001) even after adjusting for ethnicity and in fact increased the risk of carriers of the *DD* genotype from 2.34 to 2.69 for the codominant model and 2.49 for the recessive model implicating that it as an additional nonmodifiable risk factor for CHD.

The genotype frequencies reported in this cohort were found to be similar to other reports. For example, the observed frequencies for the *APOB* signal peptide polymorphism (D : 0.23, I : 0.77) for the control group (*n* = 363) were similar to those reported in other populations [26, 30, 46, 47]. The observed frequencies for the CHD patients (*n* = 372) (D : 0.32, I : 0.68) were also similar to those for the CHD patients reported for other populations [48]. The strong association and significant increase in developing CHD for the *DD* genotype carriers in the Kuwaiti population strongly suggest their role as a “risk” allele in which its contribution to the development of arthrosclerosis may be independent of other risk factors. The effect of ethnicity of the genotype distribution and its relation to CHD risk in the Kuwaiti population were evaluated by stratification analysis (Supplementary Table 1) which confirmed the need for adjusting for ethnicity in the multivariate analysis. A more than two and a half fold increase in developing CHD for the *DD* genotype remained even after adjusting for ethnicity and other risk factors including age, sex, BMI, smoking, and history of increased lipid levels. The *APOB* rs11279109 polymorphism and its association with lipid levels have demonstrated its effect on plasma lipid levels implicating the *I* allele in decreasing TG levels [26]. This further supports the “risk” role of the D allele in increasing the risk for CHD. Therefore, it is strongly recommended that this polymorphism is tested when estimating the genetic risk score for CHD.

The limitations in this study was mainly related to the sample size although a power > 80% at alpha = 0.05 was achieved (assuming an average OR of 2.5 and minor allele frequency of 7%). However, further support to the findings reported in the cohort is warranted. Other limitations included lack of actual data on lipid profile, sugar, and blood pressure values as the patient's profiles were normal as a result of being under medication for a long period of time. However, medical history was recorded from their files which made it possible to assess the risk factors using multivariate analysis. Another limitation was unavailable serum apoB protein levels.

## 5. Conclusion

Several studies have attempted to investigate the association of various genetic markers at several gene loci with the pathogenesis of CHD in different populations and ethnic groups. Studies investigating the possible association of various *APOB* polymorphisms with specific metabolic disorders or syndromes reported inconsistent findings as well as some conflicting conclusions, very likely as a result of ethnicity. The results presented revealed a statistically significant association of the *APOB* rs11279109 polymorphisms with the increased risk to develop CHD among the Kuwaiti population which has not been reported previously. This encourages the need to further assess the role of this SNP and identify the role of other SNPs at different loci in the pathogenesis of CHD independent of the known common risk factors that are specific in the ethnic group of the studied population.

## Figures and Tables

**Table 1:** Distribution of common risk factors analyzed between the CHD patients and controls in the Kuwaiti population (*n* = 735).

Variable	CHD patients *n* = 371 *n* (%)	Controls *n* = 363 *n* (%)
Sex		
Male	246 (66.1)	228 (62.8)
Female	126 (33.9)	135 (37.2)
Age (years)		
<40	38 (10.2)	45 (12.4)
40–49	73 (19.6)	79 (21.8)
50–59	103 (27.7)	118 (32.5)
≥60	158 (42.5)	121 (33.3)
Mean ± SD	55.9 ± 13.0	54.2 ± 12.8
BMI (kg/m^2^)		
<25	76 (20.4)	67 (18.8)
25–<30	131 (35.2)	133 (37.4)
≥30	165 (44.4)	156 (43.8)
Mean ± SD	29.6 ± 6.1	30.1 ± 6.9
Ethnicity^∗^		
Arab	156 (41.9)	173 (48.1)
Bedouin Arab	95 (25.5)	18 (5.0)
HU	24 (6.5)	92 (25.6)
Iranian	97 (26.1)	77 (21.4)
Smoking status^∗^		
Nonsmoker	252 (67.9)	338 (93.1)
Ex-smoker	57 (15.4)	3 (0.3)
Current smoker	62 (16.7)	22 (6.1)
Medical history of hypertension^∗^		
Yes	205 (55.1)	157 (43.3)
Medical history of high cholesterol^∗^		
Yes	172 (46.2)	102 (28.1)
Medical history of high triglycerides		
Yes	59 (15.9)	33 (9.1)
Medical history of diabetes mellitus^∗^		
Yes	197 (53.0)	62 (17.1)
Family history of cardiac diseases		
Yes	103 (27.7)	66 (18.2)

^∗^ *P* values were <0.01 and were generated by chi-square test. HU: Heterogeneous population whose mothers are not of Arab origin.

**Table 2:** Genotype distribution of the *APOB* signal peptide polymorphism among the CHD patients (*n* = 372) and controls (*n* = 363) sampled in this study.

*APOB* signal peptide	CHD patient (*n* = 372) *n* (%)	Controls (*n* = 363) *n* (%)	Total (*n* = 735) *n* (%)
II	193 (51.9)	221 (60.9)	414 (56.3)
ID	118 (31.7)	117 (32.2)	235 (32)
DD	61 (16.4)	25 (6.9)	86 (11.7)

**Table 3:** Association of the risk factors with the *APOB* signal peptide polymorphism in the Kuwaiti population investigated in this study (*n* = 735).

Variable	Genotypes			^∗^ *P* value
	*APOB* signal peptide polymorphism			
	II	ID	DD	
	*n* = 414	*n* = 235	*n* = 86	
	*n* (%)	*n* (%)	*n* (%)	
Sex				0.692
Male	264 (55.7)	151 (31.9)	59 (12.4)	
Female	150 (57.5)	84 (32.2)	27 (10.3)	
BMI (kg/m^2^)				0.511
<25	77 (53.8)	44 (30.8)	22 (15.4)	
25–<30	145 (54.9)	88 (33.3)	31 (11.7)	
≥30	190 (59.2)	98 (30.5)	33 (10.3)	
Ethnicity				0.015
Arab	192 (58.4)	102 (31.0)	35 (10.6)	
Bedouin Arab	51 (45.1)	38 (33.6)	24 (21.2)	
HU	71 (61.2)	38 (32.8)	7 (6.0)	
Iranian	98 (56.3)	56 (32.2)	20 (11.5)	
Medical history of T2DM				0.803
No	269 (56.5)	154 (32.4)	53 (11.1)	
Yes	145 (56.0)	81 (31.3)	33 (12.7)	
Medical history of hypertension				0.986
No	211 (56.6)	119 (31.9)	43 (11.5)	
Yes	203 (56.1)	116 (32.0)	43 (11.9)	
Medical history of high cholesterol				0.616
No	264 (57.3)	147 (31.9)	50 (10.8)	
Yes	150 (54.7)	88 (32.1)	36 (13.2)	
Medical history of high triglycerides				0.637
No	358 (55.7)	209 (32.5)	76 (11.8)	
Yes	56 (60.9)	26 (28.2)	10 (10.9)	
Family history of cardiac diseases				0.814
No	316 (55.8)	182 (32.2)	68 (12.0)	
Yes	98 (58.0)	53 (31.3)	18 (10.7)	

^∗^ *P* values are generated by chi-square test. T2DM: type 2 diabetes mellitus; HU: heterogeneous population whose mothers are not Arabs, or whose ancestry is unidentified.

**Table 4:** Significant independent risk factors associated with CHD selected by the multivariate logistic regression analysis (CHD patients = 372 and controls = 363).

Variables	Odds ratio (adjusted)	^a^95% CI
*APOB* signal peptide polymorphism		
II (reference group)	1.00	
ID	1.14	0.77–1.70
DD	2.43	1.34–4.41
Smoking status	6.69	3.93–11.39
Ever smoked^∗^		
Medical history of diabetes mellitus	5.73	3.77–8.71
Yes^∗^		
Medical history of high cholesterol	1.85	1.24–2.76
Yes		
Family history of cardiac diseases	1.69	1.08–2.63
Yes		

^a^95% CI = 95% confidence interval for odds ratio. ∗ indicated significance of <0.001. Included variables were sex, age, ethnicity, BMI, smoking status, medical history of diabetes mellitus, hypertension, high cholesterol, high TGs, family history of cardiac diseases, and signal peptide polymorphism.

**Table 5:** Statistical comparison of *APOB* rs11279109 genotype distribution (*n* = 668) in different genetic models between Kuwaiti CHD patients and controls after controlling for age, sex, BMI, ethnicity, smoking status, and medical history of hypercholesterolemia.

Model	Controls (*n* = 336)	CHD patients (*n* = 332)	OR (95% CI)
Codominant			
II	204 (61.4%)	174 (51.8%)	1
ID	104 (31.3%)	108 (32.1%)	1.24 (0.88–1.76)
DD	24 (7.2%)	54 (16.1%)	2.69 (1.57–4.61)
Dominant			
II	204 (61.4%)	174 (51.8%)	1
ID + DD	128 (38.6%)	162 (48.2%)	1.51(1.1-2.08)
Recessive			
II + ID	308 (92.8%)	282 (83.9%)	1
DD	24 (7.2%)	54 (16.1%)	2.49 (1.48–4.2)
Additive	332 (30.4%)	336 (68.8%)	1.5 (1.19–1.89)

CHD: coronary heart disease; OR: odds ratio; CI: confidence interval. *P* value for all models was less than 0.01.

## Abbreviations

ANOVA: Analysis of variance
APOB: Apolipoprotein B gene
APOB-SP: Apolipoprotein B signal peptide
ApoE: Apolipoprotein E gene
BMI: Body mass index
CABG: Coronary artery bypass grafting
CHD: Coronary heart disease
DALYs: Death and disability-adjusted life years
DM: Diabetes mellitus
DNA: Deoxyribonucleic acid
GWAS: Genome-wide association studies
HD: Heart disease
HDL: High-density lipoprotein
HDL-C: High-density lipoprotein cholesterol
HC: Hypercholesterolemia
HTG: Hypertriglyceridemia
HWE: Hardy-Weinberg equilibrium
LDL: Low-density lipoprotein
LDL-C: Low-density lipoprotein cholesterol
mRNA: Messenger ribonucleic acid
PCR: Polymerase chain reaction
PTCA: Percutaneous transluminal coronary angioplasty
SEM: Standard error of the mean
SNPs: Single nucleotide polymorphisms
SPSS: Statistical Package for the Social Sciences
TC: Total cholesterol
TG: Triglycerides
T2DM: Type 2 diabetes mellitus
VLDL: Very low-density lipoproteins
VNTR: Variable number tandem repeats
UNI ANOVA: Univariate analysis of variance
UV: Ultraviolet rays.

## Data Access

The authors welcome any request for additional data and documentations regarding the results from this study. Genetic data may be made available; however, no identifiers will be provided as per patient rights for confidentiality.

## Supplementary Materials

Supplementary materials Table S1. Stratification analysis of the genotype distribution for the *APOB* signal peptide polymorphisms between CHD and controls stratified according to sub-ethnicity among the studied Kuwaiti population (*n* = 668).
