# Supplementary material for: Increased Risk of the APOB rs11279109 Polymorphism for CHD among the Kuwaiti Population
Source: Dis Markers. 2017 Dec 6;2017:6963437. doi: 10.1155/2017/6963437 (PMC5737435; doi:10.1155/2017/6963437)
Supplement: Supplementary materials — Table S1. Stratification analysis of the genotype distribution for the APOB signal peptide polymorphisms between CHD and controls stratified according to sub-ethnicity among the studied Kuwaiti population (n = 668). [file 6963437.f1.pdf]

Table S1: Stratification analysis of the genotype distribution for the *APOB* signal peptide polymorphisms between CHD and controls stratified according to sub-ethnicity among the studied Kuwaiti population (n=668).

Sub-Ethnicity	Co-dominant	Controls (n=336)		CHD Patients (n=332)		OR (95% CI)
		n	%	n	%	
**Arab**		**168**		**152**		
**(n= 320)**	*II*	101	60	86	56.5	1
	ID	50	30	50	33	1.2 (0.7 – 2.03)
	DD	17	10	16	10.5	1.06 (0.5 – 2.2)
**Bedouin Arab***		**18**		**95**		
**(n= 113)**	*II*	15	83	36	38	1
	ID	3	17	35	37	4.9 (1.2 – 18.6)
	DD	0	0	24	25	-
**Iranian**		**77**		**97**		
**(n= 174)**	*II*	44	58	54	55	1
	ID	29	37	27	28	0.8 (0.4 – 1.6)
	DD	4	5	16	17	4.03 (1.1 – 13.9)
**Heterogenous**		**100**		**27**		
**(HU)****	*II*	61	61	16	59	1
**(n= 127)**	*ID*	35	35	6	22	0.78 (0.2 – 2.3)
	DD	4	4	5	19	7.09 (1.5 – 33.2)

* p-value for all models was less than 0.01; ** p-value for all models was less than 0.05
